# Cyclosporine A alleviates colitis by inhibiting the formation of neutrophil extracellular traps via the regulating pentose phosphate pathway

**DOI:** 10.1186/s10020-023-00758-8

**Published:** 2023-12-13

**Authors:** Chenjing Xu, Ziping Ye, Wenyu Jiang, Shu Wang, Hongjie Zhang

**Affiliations:** Department of Gastroenterology, The First Affiliated Hospital of Nanjing Medical University, Nanjing Medical University, Nanjing, Jiangsu China

**Keywords:** Ulcerative colitis, Cyclosporine A, Neutrophil extracellular traps, P53, Pentose phosphate pathway, Glucose-6-phosphate dehydrogenase, Reactive oxygen species

## Abstract

**Background:**

The aberrant formation of neutrophil extracellular traps (NETs) has been implicated in ulcerative colitis (UC), a chronic recurrent intestinal inflammation. Cyclosporine A (CsA) is now applied as rescue therapy for acute severe UC. In addition, it has been certained that CsA inhibits the formation of NETs in *vitro* and the mechanism of which was still vague. The study aimed to explore the mechanism CsA inhibits the NETs formation of colitis in vivo and in *vitro*.

**Methods:**

NETs enrichment in clinical samples was analyzed using databases from Gene Expression Omnibus and verified in our center. Dextran sulfate sodium (DSS)-induced acute colitis mice model was used to investigate the effect of CsA on NETs of colonic tissue expression. To clarify the mechanism, intracellular energy metabolites were examined by Liquid Chromatograph Mass Spectrometer, and reactive oxygen species (ROS) levels were examined by fluorescence intensity in neutrophils treated with CsA after LPS stimulation. The transcriptional level and activity of G6PD of neutrophils were also assessed using qRT-PCR and WST-8. RNA Sequencing was used to detect differentially expressed genes of neutrophils stimulated by LPS with or without CsA. The expression levels of related proteins were detected by western blot.

**Results:**

NETs enrichment was especially elevated in moderate-to-severe UC patients compared to HC. NETs expression in the colon from DSS colitis was decreased after CsA treatment. Compared with neutrophils stimulated by LPS, NETs formation and cellular ROS levels were decreased in LPS + CsA group. Cellular ribulose 5-phosphate and NADPH/NADP + related to the pentose phosphate pathway (PPP) were reduced in LPS + CsA group. In addition, CsA could decrease G6PD activity in neutrophils stimulated with LPS, and the results were further verified by inhibiting G6PD activity. At last, P53 protein was highly expressed in LPS + CsA group compared with the LPS group. Intracellular G6PD activity, ROS level and NETs formation, which were downregulated by CsA, could be reversed by a P53 inhibitor.

**Conclusion:**

Our results indicated CsA could alleviate the severity of colitis by decreasing the formation of NETs in *vivo*. In *vitro*, CsA reduced ROS-dependent NETs release via downregulating PPP and cellular ROS levels by decreasing G6PD activity directly by activating the P53 protein.

**Supplementary Information:**

The online version contains supplementary material available at 10.1186/s10020-023-00758-8.

## Introduction

Ulcerative colitis (UC) is a recurrent and incurable disease, and its etiology and pathogenesis are still undetermined (Ungaro et al. 2017). The immune dyshomeostasis is one of the essential phenomena existing in UC and could participate in pathogenesis (Boland et al. 2020; de Souza and Fiocchi 2016). Innate immunology, including the function of macrophages and neutrophils, has been more activated (Corridoni et al. 2018). Neutrophils have been considered to represent the severity of UC patients and are the histologic activity marker (Pai et al. 2020). It is manifested as neutrophil infiltration between epithelial cells, cryptitis, crypt abscesses, and even formation of mucosal ulceration (Ungaro et al. 2017).

The identified forms of neutrophil death include non-inflammatory apoptosis and autophagy, inflammatory pyroptosis, necrosis and neutrophil extracellular traps (NETs) (Geering et al. 2011). NETs, a web-like structure consisting of granule proteins and chromatin, were first recognized as the antimicrobial instrument (Brinkmann et al. 2004), which could also stimulate monocytes to excrete proinflammatory cytokines and trigger dendritic cells (DCs) activation (Dinallo et al. 2019; Sangaletti et al. 2012). In the dextran sodium sulfate (DSS)-induced colitis model, the intestinal barrier was damaged by NETs via the induction of enterocyte apoptosis (Lin et al. 2020). Moreover, it has been confirmed that the expression of NETs-related proteins increased in inflammatory tissues of patients with UC (Bennike et al. 2015; Dinallo et al. 2019; Li et al. 2020). As a result, the downregulation of NETs formation might help alleviate colitis (Dinallo et al. 2019).

Cyclosporine A (CsA) has been applied to clinical treatment for autoimmune diseases like rheumatoid arthritis and UC (Kitahara and Kawai 2007; Laharie et al. 2012; Tugwell et al. 1995). It could inhibit the secretion of interleukin-2 (IL-2) from T cells and also interfere migration of DCs (Chen et al. 2004; Liu et al. 1991). Recently, it has been verified that CsA could modulate neutrophil functions such as migration and secretion of reactive oxygen species (ROS) (Lu et al. 2021). In virto, it has been found that CsA could decrease the formation of NETs (Vorobjeva et al. 2020). However, the underlying mechanisms of CsA-mediated decreasing the formation of NETs were still unclear.

At present, it has been well accepted that ROS and Ca^2+^ mobilization in neutrophils under different stimuli associated with the formation of NETs remains the predominant canonical pathway, despite the existence of non-canonical pathway of ROS-independent NETs release (Azzouz et al. 2021; Hann et al. 2020). The origin of ROS in neutrophils was mainly dependent on Nicotinamide Adenine Dinucleotide Phosphate (NADPH) oxidase (NOX) (Nguyen et al. 2017) and exposure to lipopolysaccharide (LPS) resulted in NOX-dependent or lytic NETs formation (Kraaij et al. 2016; Pieterse et al. 2018). Pentose Phosphorylation Pathway (PPP), another way of glucose oxidative decomposition, produced the main proportion of NADPH in neutrophils (Azevedo et al. 2015). PPP is essential for the release of NETs, because it plays an important role in fueling Nox with NADPH to produce an effective species of ROS and thus induce NETs (Amara et al. 2021; Britt et al. 2022). Recently, CsA was found to regulate the function of neutrophils through an increasing flux of glycolysis (Lu et al. 2021), however, one study reported that PPP maybe more important and the effect of CsA on the PPP in neutrophils still needs to be determined (Azevedo et al. 2015).

Given the essential role of NETs in the regulating immunity and intestinal barrier function, our study investigated the condition of NETs production in patients with UC using the data from GEO database and clinical samples and the influence of CsA on the formation of NETs in *vivo* and in *vitro*. We determined that CsA may alleviate colitis by decreasing the formation of NETs through regulating PPP.

## Methods and materials

### Data acquiring and possessing

RNA Sequencing (RNA-seq), microarray and clinical characteristics analyzed in this paper were obtained from the Gene Expression Omnibus (GEO) repository (https://www.ncbi.nlm.nih.gov/geo/) (GSE109142、GSE73661、GSE94648). A reference NET signature comprising 23 genes (Additional file 2: Table S1) was derived using proteins enriched in NETs released from human neutrophils and were defined as NETs-related genes (Shen et al. 2022). *Limma* package (R-4.1.2) was used to find the changes in NETs-related gene-set expression between patients with UC and healthy control (HC) (Gardinassi et al. 2017; Shen et al. 2022). *GSVA* package (R-4.1.2) was operated to calculate the enrichment score of NETs-related gene-set (NETs score) based on 23 major component genes and study the relationship between NETs score and severity of UC (which were evaluated by histologic severity score (grade I-VI) (Haberman et al. 2019), Mayo endoscopic score (MES) and Mayo score).

### Patients and sampling

The Ethics Committee of The First Affiliated Hospital of Nanjing Medical University approved the study. Participants have submitted informed consent. The patients whose Mayo scores ≥ 6 considering the moderate and severe activity of clinical symptoms, endoscopic features and inflammatory markers, were enrolled in this study. The patients with a history of hypertension, diabetes, autoimmune disease, vascular disease, surgery, and receiving biologics (anti-TNF-α agents and vedolizumab) treatment were excluded. Six pairs of tissue biopsies and 10ml EDTA-anticoagulated blood samples were obtained from six pairs of UC patients and HC for further study.

### Animals and grouping

The Experimental Animals Ethics Committee of Nanjing Medical University has approved this study. Male C57BL/6 J mice (7–8 weeks old, 18–20 g, specific pathogen-free) were purchased from Vital River Laboratory Animal Technology and housed under standardized conditions, which is 22 ± 2℃ and 12 h light/dark cycle. To induce acute colitis, mice were supplied with 3% DSS in germ-free water to drink freely for seven days. In the CsA-treated group, mice were injected with dissolvent CsA (0.9% NaCl solution containing 0.7% ethanol and 1.3% polyoxyethylene castor oil) via intraperitoneal route daily during 7-day 3% DSS solution induction. Mice were sacrificed on day 7. Then colon tissues were dissected and preserved at -80℃ for further analysis. To assess the severity of histopathology, the rest of tissues were fixed in 50% ethanol/5% acetic acid in ddH2O and embedded into paraffin (Bialkowska et al. 2016), which were cut into 3 μm pieces for haematoxylin and eosin [H&E] staining. The criteria of mucosal inflammation were referred to as described previously (Arranz et al. 2012).

### Peripheral blood neutrophil isolation and G6PD activity examination

Neutrophils from peripheral blood were isolated using human neutrophils isolation kit [TBD science] according to instruction. Then G6PD(rate-limited enzyme of PPP) activity of unstimulated neutrophils or neutrophils (1 × 10^6^ per well) stimulated by 2.5 μg/ml LPS [Sigma-Aldrich] with or without 100 nM CsA for 30 min were measured using G6PDH Activity Assay Kit with WST-8[Beyotime] (Zhang et al. 2022) and normalized by protein quantification.

### Peripheral blood NETs incubation, visualization, and quantification

Isolated neutrophils were resuspended in RPMI 1640 medium with 1% Penicillin–Streptomycin Solution [Gibco] and stabilized at 37℃ and 5% CO_2_ for 1 h. After that, cells (2 × 10^4^ cells per well) were distributed in black 96-well plates with clear bottoms coated with Poly-L-Lysine [Beyotime] and stimulated with 2.5 μg/ml LPS for 3 h. For treatment, 100 nM Cyclosporine A [CsA, MCE] in RPMI was added 5 min after LPS stimulation, and 10 μM 6-Aminonicotinamide [6-AN, MCE] of an inhibitor of G6PD or 20 μM Pifithrin-α hydrobromide [PFT-α, MCE] of an inhibitor of P53 was administered simultaneously.

For visualization, cells in 96-well plates were incubated with 0.2 μM Sytox green [Thermo Fisher Scientific] stained for detecting dsDNA of dead cells and 10 μg/ml Hoechst 33342 [Solarbio] stained for live cells when stimulation began (White et al. 2017). After 3 h, NETs could be directly visualized on an ECLIPSE Ti2 Inverted Microscope [Nikon]. For quantification, the fluorescence intensity of Sytox green and Hoechst 33342 were scanned on a BioTek Cytation 1 cell imaging multimode reader [Agilent] with excitation/emission of 488/525 nm and 350/461 nm, respectively. To compare NETs formation among patients, 0.5% TritonX-100 (v/v) in RPMI 1640 was set as the positive control.

### ROS measurement

Isolated neutrophils were resuspended in Phosphate Buffered Saline (PBS) with 1% Penicillin–Streptomycin Solution [Gibco] and labelled with ROS probe utilizing 5 μM DCFH-DA [MCE]. Then cells were seeded in black 96-well plates with clear bottoms (2 × 10^4^ cells per well). When stimulation began, the fluorescence intensity of DCFH-DA was measured and recorded every 10 min for 1 h on a BioTek Cytation 1 cell imaging multimode reader [Agilent] with excitation/emission of 488/525 nm.

### Immunofluorescence

For tissue samples from humans and mice, the sections were baked, deparaffinized, rehydrated and performed antigen retrieval in Citrate Antigen Retrieval solution [Beyotime]. The sections were stained with anti-neutrophil elastase (NE) mouse monoclonal antibody [1:100, Abcam], anti-citrullinated histone h3 (citH3) rabbit polyclonal antibody [1:100, Abcam], anti-NE rabbit polyclonal antibody [1:100, ABclonal] at 4℃ overnight. Then the sections were incubated Fluorescein (FITC)–conjugated Goat Anti-Mouse IgG (H + L) [1:100, Proteintech], Cy3–conjugated Goat Anti-Rabbit IgG (H + L) [1:50, Proteintech] at 37℃for 1 h. For antibodies from the same origin, Three-color Fluorescence Kit [Recordbio] was applied to the label. Then the sections and slides were incubated with DAPI [Beyotime] and covered with antifade mounting medium [Beyotime]. The images were obtained on a STELLARIS STED laser confocal microscope [Leica].

### Western blotting

Tissues obtained from the mice and neutrophils treated by LPS (2.5 μg/ml), with or without CsA (100 nM) or PFT-α(20 μM) for 3 h were lysed in RIPA buffer [Beyotime] mixed with protease inhibitor cocktail [CST] on ice for 30 min. The protein concentration in the supernatant was measured using BCA Protein Quantification Kit [Beyotime]. After blended with loading buffer [Beyotime] and denatured, the extracted protein was separated on an SDS–polyacrylamide gel and transferred onto a polyvinylidene difluoride membrane [Millipore]. Then the gels were blocked with 5% milk in 0.1% Tween 20 in Tris-buffered saline (v/v, TBST) solution at room temperature for 1 h, followed by incubation with anti-NE rabbit polyclonal antibody [1:1000, ABclonal], anti-citH3 rabbit polyclonal antibody [1:1000, Abcam], anti-β-actin mouse monoclonal antibody [1:10,000, Proteintech], anti-P53 mouse monoclonal antibody [1:1000, Abcam], and anti-G6PD mouse monoclonal antibody [1:1000, Proteintech] at 4℃ overnight respectively. After incubated with Peroxidase-conjugated AffiniPure Goat Anti-Rabbit IgG (H + L) [1:10,000, Jackson ImmunoResearch], Peroxidase-conjugated AffiniPure Goat Anti-Mouse IgG (H + L) [1:10,000, Jackson ImmunoResearch] separately, the images were detected by Chemiluminescence Imaging System [Tanon] using BeyoECL plus [Beyotime].

### Reverse transcription-polymerase chain reaction analysis

The tissues from mice and cells were lysed in Trizol [Thermo Fisher Scientific]. Then RNA was extracted and reversed into cDNA for further reaction using HiScript Reverse Transcriptase [vazyme]. The primer sequences for qRT-PCR were shown in the Additional file 3: Table S2.

### Cell metabolisms quantification assay

Neutrophils (1 × 10^7^) in each group were stimulated by LPS with or without CsA for 30 min. Then cells were harvested in 500 μl Methanol: acetonitrile: H_2_O (2:2:1, v/v/v) and vortexed for 60 s, followed by ultrasound for 30 min twice. Metabolites were separated using ultra-high-performance Liquid Chromatography (Agilent 1290 Infinity LC). The mobile phases comprised solvent A (10 mM ammonium acetate) and solvent B (Acetonitrile). The sample (2ul) was injected in the column with a temperature of 45℃ and a flow rate of 0.3ml/min. The gradient was programmed as follows: 0–18 min from 90 to 40% B; 18–18.1 min, from 40 to 90% B; 18.1–23 min, 90% B. Then the products were analyzed utilizing 5500 QTRAP Liquid Chromatography-Mass Spectrometer (LC–MS) coupled with an electronic spray ionization source, which was operated on negative mode. Data acquisition was performed in multiple reaction monitoring modes (MRM). Quantification was performed using MultiQuant 3.02.

For NADPH assay, neutrophils (1 × 10^7^) in each group were stimulated by LPS with or without CsA for 30 min and then harvested with 4℃ PBS. NADPH Assay Kit [Beyotime] was used to detect.

### RNA Sequencing

Neutrophils (1 × 10^7^) were stimulated by LPS (2.5ug/mL) with or without CsA (100 nM) for 30 min, and cell samples were collected by Trizol. After total RNA was extracted from the samples, mRNA was isolated and fragmented. The mRNA fragments were taken as templates. The first and the second cDNA strand was synthesized. After purification, the target-size fragments were recovered by agarose gel electrophoresis for PCR amplification. The constructed library used the DNBSEQ platform (BGI-Shen Zhen) and differential expression analysis was performed using the DESeq2.

### Statistic and analysis

All results were representative of at least three independent experiments. Data were shown as the mean ± standard error of the mean [SEM]. Statistical analysis was performed using SPSS version 24.0 [IBM SPSS Statistics, USA] and GraphPad Prism [Prism 8 software, USA]. For comparing two groups, paired or unpaired Student t-tests were used. One-way analysis of variance was used for multiple comparisons, followed by Tukey’s post-hoc test. In some cases, results have normalized to that at the beginning before comparison.

## Results

### Elevated NETs expression in UC patients

**Fig. 1:**
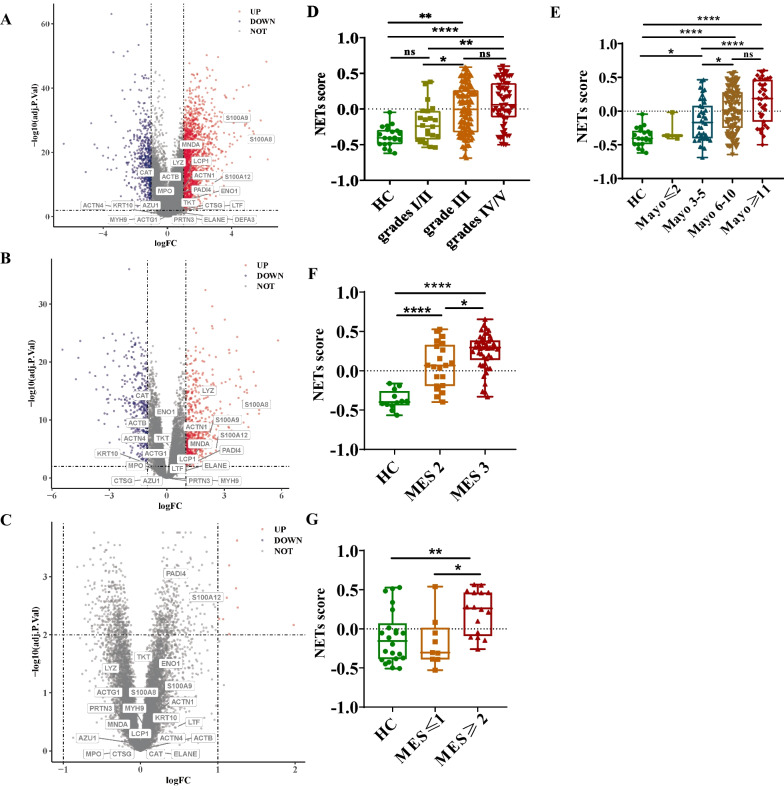
NETs enrichment in UC patients. **A** Volcano plot of differential gene expression labeled with NETs gene-set in rectum between UC patients and HC. **B** Volcano plot of differential gene expression labeled with NETs gene-set in colon between UC patients and HC. **C** Volcano plot of differential gene expression labeled with NETs gene-set in whole blood between UC patients and HC. **D** NETs score in rectum of patients evaluated by histologic severity score (*P < 0.05 for patients with grade I/II vs grade III, **P < 0.01 for HC vs patients with grade III, **P < 0.01 for patients with grade I/II vs grade IV/V, ****P < 0.0001 for HC vs patients with grade IV/V). **E** NETs score in rectum of patients evaluated by Mayo score. **F** NETs score in colon of patients evaluated by MES. **G** NETs score in whole blood of patients evaluated by MES

To evaluate the condition of NETs formation, RNA-seq and microarray of bowel tissues and whole blood from UC patients and HC were used to find differential expression followed by labelling NETs-related gene-set. In RNA-seq of rectal and colonic tissues from UC patients (GSE109142, GSE73661), nearly half of the genes in the NETs-related gene-set were upregulated, especially *S100A9*、*S100A8* and *S100A12* (Fig. 1A&B). However, no significantly upregulated genes were identified in gene expressions of microarray in whole blood (GSE94648) between UC patients and HC (Fig. 1C).

Then we analyzed the relationship between NETs scores and the disease severity of UC patients. In GSE109142, NETs scores of the rectal biopsy were correlated with histologic severity scores. NETs scores were higher in UC patients with moderate and severe inflammation (grade III-V) compared with HC. However, there was no difference between patients with mild histological features (grade I/II) and HC (Fig. 1D). Likewise, the NETs score was significantly increased in patients with moderate and severe clinical disease activity evaluated by the Mayo score compared to HC (Fig. 1E). Similar results were confirmed in colonic tissues from GSE73661. Patients with MES of 2 or more had higher NETs scores compared with HC (Fig. 1F, G). Thus, it was identified that NETs scores were increased in patients with UC relative to disease severity.

**Fig. 2:**
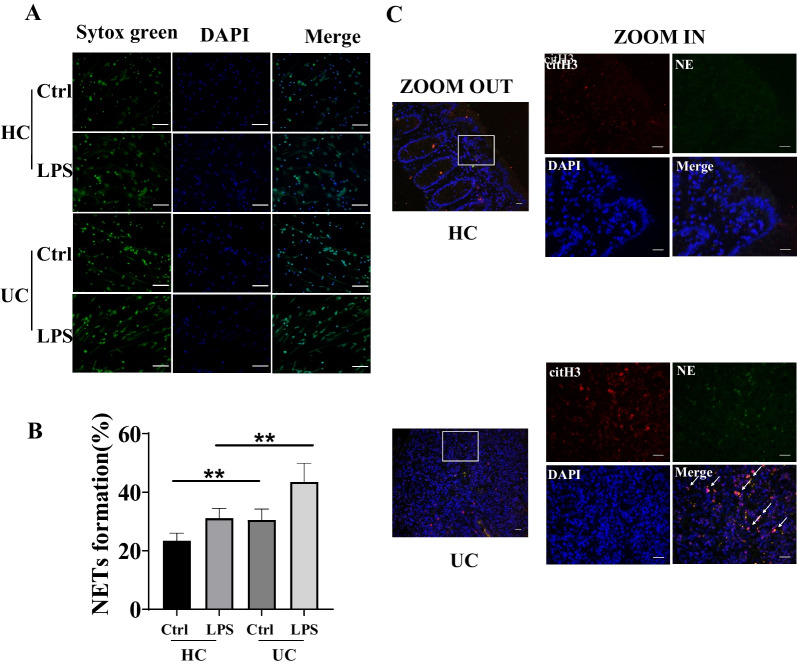
NETs expression in peripheral blood and colonic tissue from UC patients and HC. **A** Immunofluorescence of NETs with or without stimulation with LPS in neutrophils isolated from UC patients and HC; Green: Sytox green, Blue: Hoechst 33342. **B** Quantification of NETs with or without stimulation with LPS in neutrophils isolated from UC patients and HC (% of 0.5% Triton X-100 treated group, **P < 0.01 for unstimulated neutrophils from HC vs UC and neutrophils stimulated by LPS from HC vs UC). **C** Immunofluorescence of NE (green)、citH3 (red) and nuclear stained with DAPI (blue) in colonic tissue from UC patients and HC

Then clinical samples in our center were gathered to be further proven. We effectively used Sytox green to detect dsDNA of NETs in peripheral blood neutrophils and Hoechst 33342 staining for live cells. Although interference of dsDNA produced by other forms of cell death cannot be ruled out, the amount of dsDNA produced by other forms of cell death in the peripheral blood assay is relatively low compared with the amount produced by NETs death. The ratio of the intensity of Sytox Green and Hoechst 33342 was upregulated in neutrophils of peripheral blood stimulated with LPS in UC patients than HC. Moreover, it was both statistically significant for unstimulated neutrophils from HC vs UC and neutrophils stimulated by LPS from HC vs UC (P < 0.01) (Fig. 2A, B). Also, the level of NETs-related proteins, NE and citH3, were elevated in colonic tissue from UC patients compared with that from HC (Fig. 2C). Taken together, we confirmed that NETs formation is closely related to UC.

### CsA ameliorated colitis in DSS-induced colitis mice and downregulated the expressions of NETs-related proteins in the colon

**Fig. 3:**
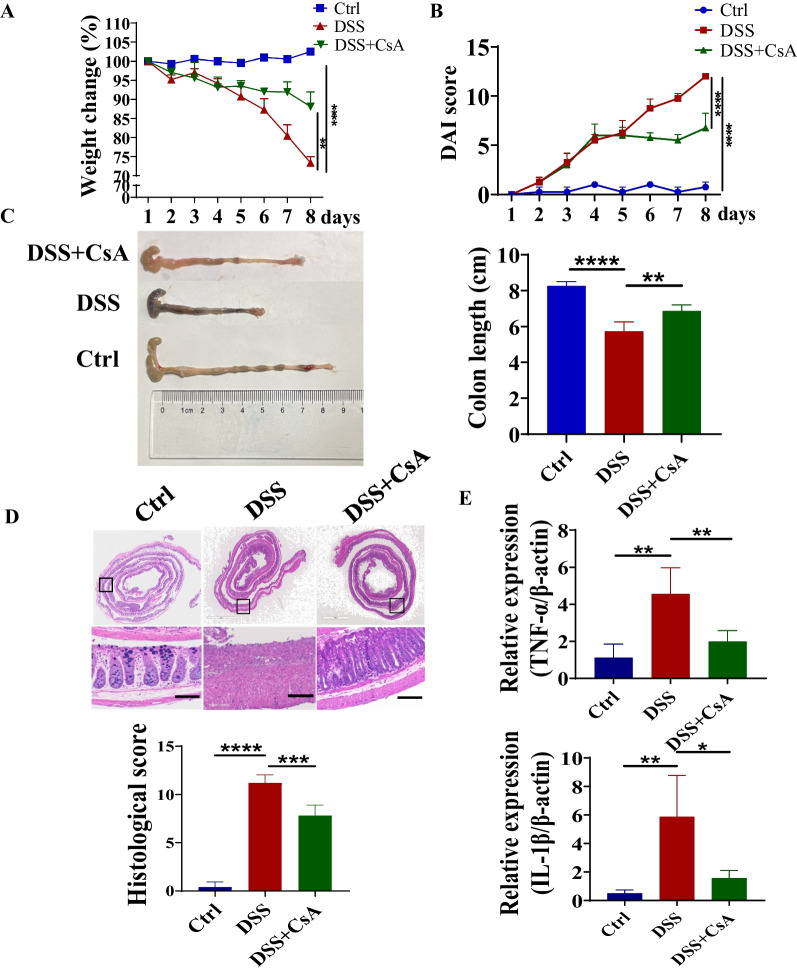
Attenuated severity of colitis in DSS-induced mice model under the treatment of CsA. **A** Percentage of weight change in mice from day 1 to day 8 (**P < 0.01 for DSS vs DSS + CsA group, ****P < 0.0001 for Ctrl vs DSS group). **B** Disease activity index scores in mice from day 1 to day 8 (****P < 0.0001). **C** Appearance and lengths of colons in mice on day 8 (**P < 0.01, ****P < 0.0001). **D** Histological feature and scores of colons in mice on day 8. **E** The transcriptional level of TNF-α and IL-1β of colonic tissue in mice on day 8 (*P < 0.05, **P < 0.01)

**Fig. 4:**
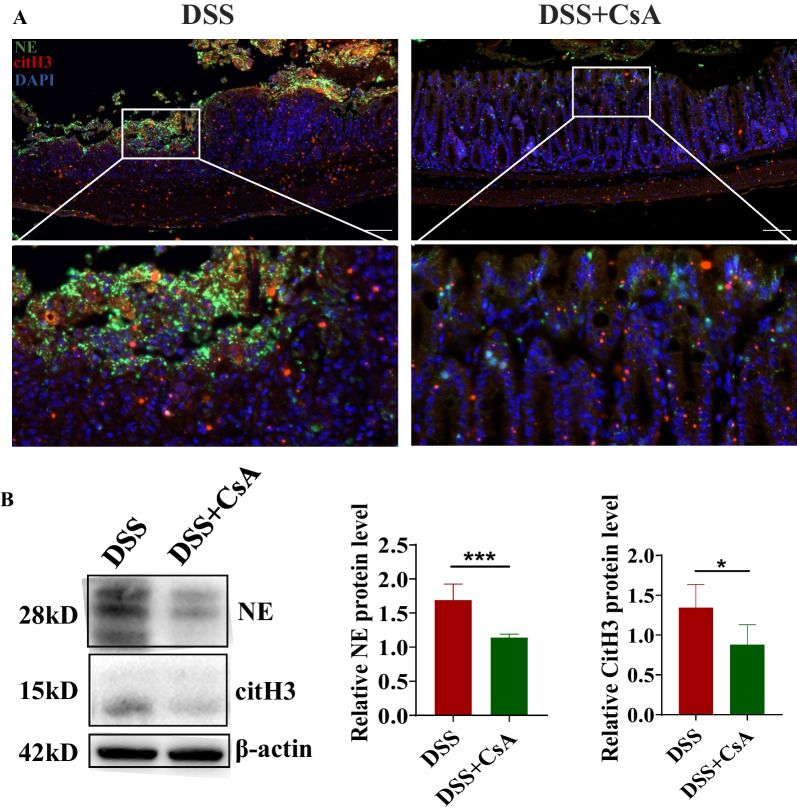
NETs expression in colon from DSS-induced mice model under the treatment of CsA. **A** Immunofluorescence of NE (green)、citH3 (red) and nuclear stained with DAPI (blue) of colon in mice on day 8. **B** Western blotting and quantification of the expression of NE and citH3 of colon in mice on day 8 (*P < 0.05, ***P < 0.001)

Since the formation of NETs was increased in colon and peripheral blood from UC patients, DSS was used to induce acute colitis mice model to explore further the effect of CsA on NETs release in intestinal tissues. CsA could decrease the percentage of weight change and disease activity index scores in DSS-induced colitis mice (Fig. 3A, B). The shortness of the colon and the severity of bloody stool were also ameliorated in colitis mice with the treatment of CsA (Fig. 3C). In addition, CsA decreased histology damage and the histological scores in colitis mice by evaluating the results of H&E staining (Fig. 3D). CsA reduced the transcriptional level of inflammatory cytokines (TNF-α and IL-1β) in colon tissues (Fig. 3E). Then the expression of NETs-related proteins was examined to evaluate NETs release in colon by immunofluorescence and further verified by western blotting. We found that levels of NE and citH3 expression were significantly downregulated in the colon from the CsA-treated group both in immunofluorescence and western blotting (Fig. 4A, B). Therefore, CsA was able to reduce the NETs release besides dampening the severity of colitis.

### CsA reduced LPS-stimulated NETs release and decreased PPP metabolism in neutrophils isolated from human peripheral blood

**Fig. 5:**
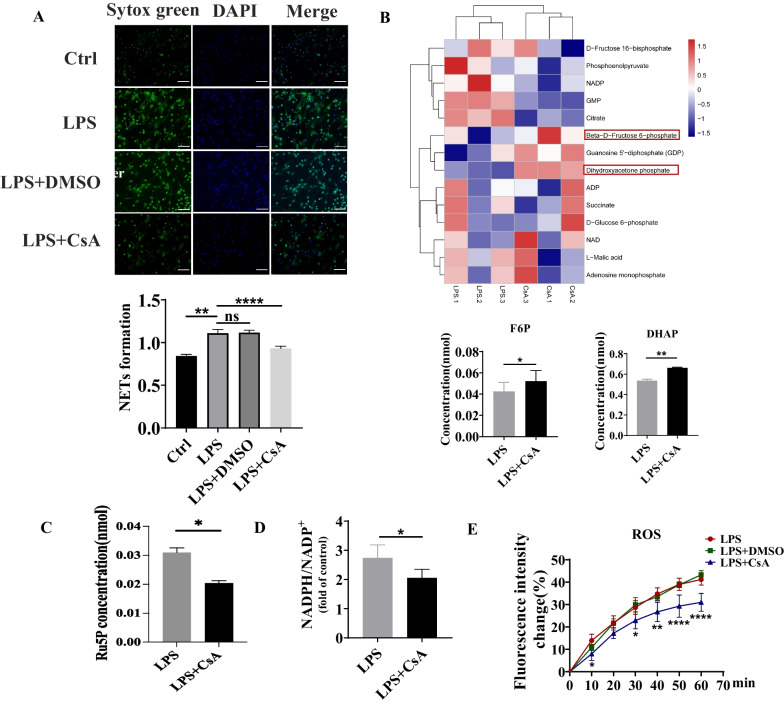
NETs expression, intracellular ROS level, and energy metabolism in neutrophils under the treatment of CsA. **A** Immunofluorescence and ratio of fluorescence intensity of Sytox green to Hoechst 33342 in neutrophils stimulated by LPS at the presence of CsA; Green: Sytox green, Blue: Hoechst 33342 (**P < 0.01 for Ctrl vs neutrophils stimulated by LPS, ****P < 0.0001 for neutrophils stimulated by LPS without CsA vs with CsA). **B**, **C** Intracellular energy metabolites in neutrophils stimulated by LPS at the presence of CsA (*P < 0.05, **P < 0.01). **D** NADPH/NADP + in neutrophils stimulated by LPS at the presence of CsA (*P < 0.05). **E** Change of intracellular ROS level in neutrophils stimulated by LPS at the presence of CsA for 1 h (*P < 0.05, **P < 0.01, ***P < 0.001 for LPS vs LPS + CsA)

It is difficult to isolate neutrophils from intestinal tissue and to directly verify the effect of CsA on NETs release, neutrophils isolated from the peripheral blood of HC were treated with CsA after LPS stimulation. The ratio of the intensity of Sytox Green and Hoechst 33342 was significantly downregulated after CsA treatment from visualization and quantification, which suggested CsA can reduce NETs release (Fig. 5A).

We next investigated the possible intrinsic mechanism of CsA in reducing NETs release. NOX, the main source of ROS, can consume NADPH to generate ROS in neutrophils, while PPP was the primary origin of NADPH. We hypothesized that the functional changes of neutrophils stimulated by CsA could be mediated by the PPP and it provided NADPH for maintaining Nox activity and ROS production. Quantitative change in the energy metabolism of neutrophils was detected by LC–MS. After being treated by CsA, the results showed that the concentration of intracellular fructose 6-phosphate (F6P) and dihydroxyacetone phosphate (DHAP) related to glycolysis significantly upregulated (Fig. 5B). In order to clarify the changes in PPP metabolism, ribulose 5-phosphate (Ru5P), the important PPP intermediates significantly downregulated in LPS + CsA group (Fig. 5C). Furthermore, the intracellular NADPH/NADP + ratio of neutrophils was detected using the NADPH Assay Kit. The results showed that compared with LPS group, the ratio of NADPH/NADP + in LPS + CsA group was decreased (P < 0.05) (Fig. 5D).

Given the importance of ROS during the process of NETs release, we assessed the level of ROS in peripheral blood neutrophils treated with CsA. ROS probe (DCFH-DA) was utilized to examine the level of intracellular ROS of neutrophils stimulated by LPS for 1 h in the presence of CsA. The results showed that compared with LPS group, there was no significant change in intensity of the ROS probe in LPS + DMSO group, but the LPS + CsA group was decreased (P < 0.0001) (Fig. 5E).

### NETs release was dependent on intracellular PPP metabolism

**Fig. 6:**
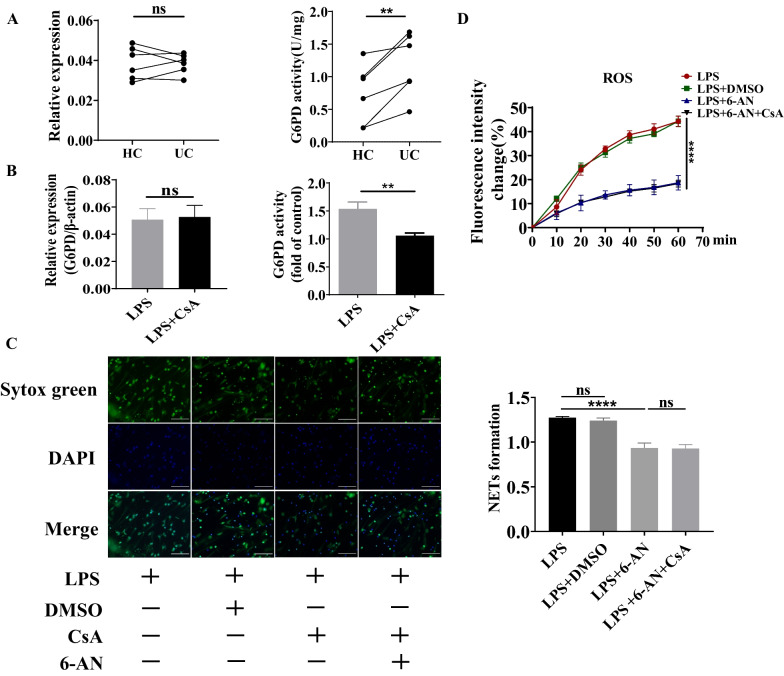
The influence of CsA on NETs expression dependent on G6PD. **A** The activity and transcriptional level of G6PD in neutrophils from UC patients and HC (ns: not significance, **P < 0.01). **B** The activity and transcriptional level of G6PD in neutrophils treated with LPS with or without CsA (ns: not significance, **P < 0.01). **C** Immunofluorescence and ratio of fluorescence intensity of Sytox green to Hoechst 33342 in neutrophils stimulated by LPS at the presence of 6-AN; Green: Sytox green, Blue: Hoechst 33342 (****P < 0.0001 for neutrophils treated with LPS vs LPS + 6-AN). **D** Change of intracellular ROS level in neutrophils stimulated by LPS in the presence of 6-AN for 1 h (ns: not significance, ****P < 0.0001 for neutrophils treated with LPS vs LPS + 6-AN)

Data are showing that G6PD, the first enzyme of the PPP, is important for ROS production and ROS-dependent NETs release (Azevedo et al. 2015). For further investigation, the change of activity of G6PD was examined. It was found that although the transcriptional level of G6PD was unaffected, the activity of G6PD was upregulated in neutrophils isolated from human peripheral blood (Fig. 6A). We have further proved that CsA could decrease the activity of G6PD in neutrophils isolated from human peripheral blood induced by LPS, but the transcriptional level of G6PD was no different under the effect of CsA (Fig. 6B). Above all, downregulated NETs release treated by CsA had a possible relationship with distinct PPP metabolism in neutrophils.

To investigate the influence of PPP during the process of NETs release, we utilized the inhibitor of G6PD, 6-AN, to treat neutrophils. It was found that NETs release was significantly reduced in LPS and 6-AN treated group compared with neutrophils stimulated by LPS alone, while there was no difference when compared with LPS, 6-AN, and CsA treatment at the same time (Fig. 6C). Turing our attention to intracellular ROS level, it was also illustrated that intracellular ROS remained unchanged after being induced by 6-AN with or without the presence of CsA (Fig. 6D). Above all, PPP was irreplaceable in the process of the effect of CsA on NETs release.

### CsA downregulated PPP metabolism and decreased NETs release by inhibiting the expression of P53

**Fig. 7:**
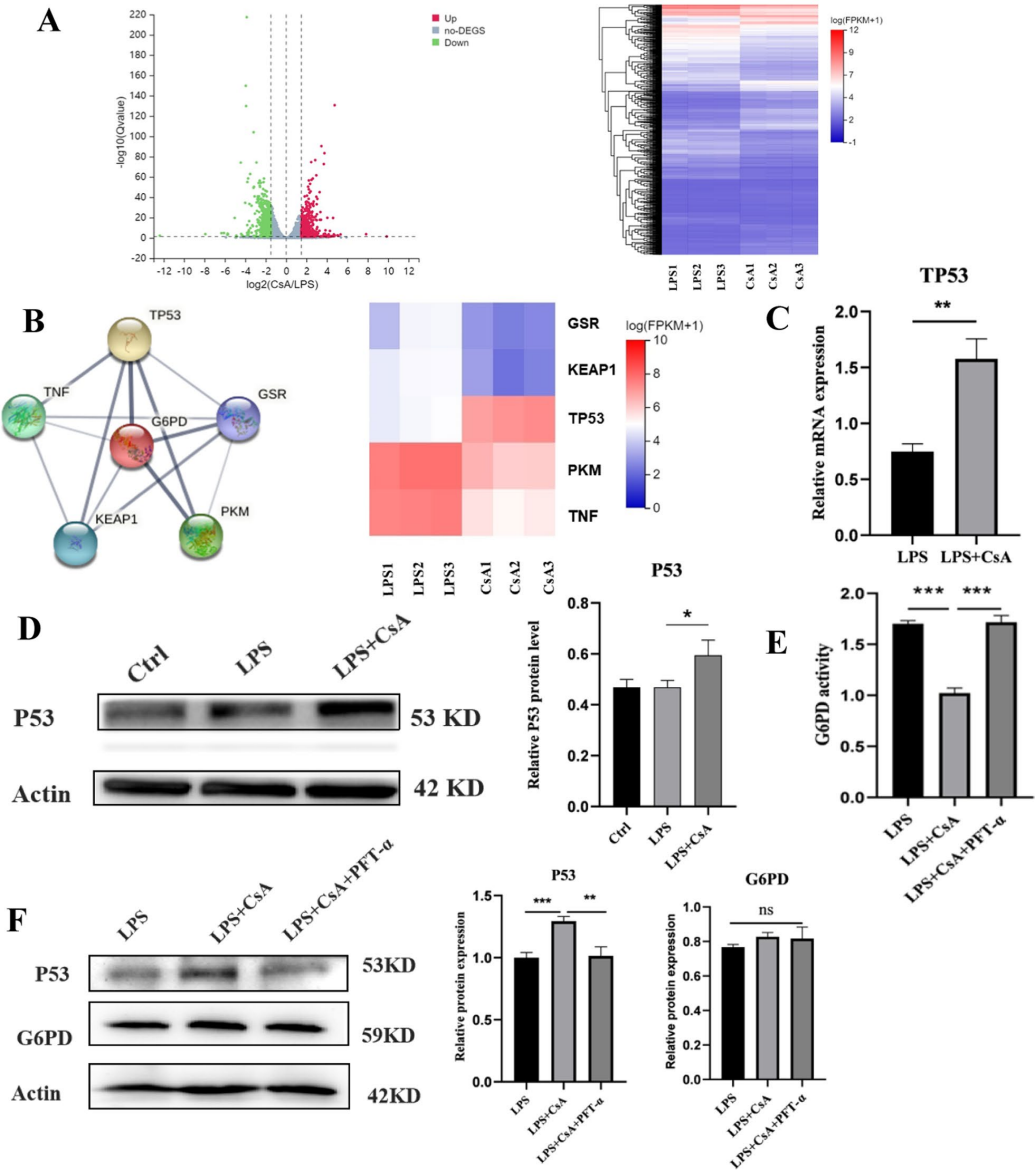
The decrease of G6PD activity but not protein level dependent on activation of P53 protein by CsA. **A** Volcano map and cluster heat map of differentially expressed genes in LPS and LPS + CsA groups (|log2FC|≥ 1.5, Qvalue ≤ 0.05). **B** Protein interaction between differentially expressed genes and G6PD and their cluster heat map. **C** TP53 transcription levels in the two groups (**P < 0.01 for neutrophils treated with LPS vs LPS + CsA). **D** Western blotting and quantification of the expression of P53 protein level in each group (*P < 0.05 for neutrophils treated with LPS vs LPS + CsA). **E** The activity of G6PD in neutrophils under the treatment of PFT-α for 3 h (***P < 0.001 for neutrophils treated with LPS + CsA vs LPS and LPS + CsA + PFT-α). **F** Western blotting and quantification of the expression of P53 and G6PD protein level in each group (ns: not significance; **P < 0.01 for neutrophils treated with LPS + CsA vs LPS + CsA + PFT-α; ***P < 0.001 for neutrophils treated with LPS vs LPS + CsA)

The RNA-seq investigated the possible mechanism of the influence of CsA on G6PD. Using the criteria of > 1.5-fold increase or decrease, 1248 genes with significantly altered expression were observed between the two groups with or without CsA (Fig. 7A). Then by consulting research and retrieval in STRING, the protein–protein interaction database, the top five genes related to G6PD were targeted. Among these genes, P53 was found to interact with G6PD the most tightly. The heatmap showed the relative expression of five genes in RNA-seq between the CsA group and the control group (Fig. 7B). The transcriptional level of TP53 was verified to be elevated in the CsA group by qRT-PCR (Fig. 7C). We also confirmed the activation of the P53 protein with the existence of CsA by Western blot (Fig. 7D). To further elucidate whether CsA reduces the activity of G6PD by activating P53 protein and its effect on the level of G6PD protein expression, the P53 inhibitor was utilized. It was shown that compared with the LPS group, the G6PD activity in the LPS + CsA group was decreased, which could be reversed with the presence of PFT-α (Fig. 7E). However, no significant difference between the groups could be observed in G6PD protein expression (Fig. 7F). Lastly, to investigate whether CsA reduced the ROS generation and NETs release based on P53 inhibition, PFT-α was utilized to find that it reversed the inhibitory effect of CsA on ROS and NETs release (Additional file 1: Fig. S1A, B). In general, CsA might reduce the G6PD activity by activating P53 protein, reducing ROS generation in the PPP metabolic pathway, thereby reducing ROS-dependent NETs release.

## Discussion

CsA has been utilized as rescue therapy for patients with acute severe UC (Dulai and Jairath 2018; Song et al. 2021). In addition, there was no difference in the 3-year survival and colectomy rate evaluation between the UC therapy of CsA and infliximab (Laharie et al. 2012). As described in the previous study, successful regulation of neutrophils function, which plays a crucial role in the pathogenesis and prognosis of UC, like apoptosis and migration, was indispensable in CsA-responsive patients (Fournier and Parkos 2012; Lu et al. 2021). Nevertheless, it remains unclear whether CsA could modulate NETs release in colitis. In this study, we explored the possible mechanisms of CsA in the UC treatment and observed that CsA can inhibit the formation of NETs. CsA ameliorated histology damage and the histological scores, decreased inflammatory cytokines (TNF-α and IL-1β) expression and downregulated NE and citH3 expression of NETs-related proteins in DSS-induced colitis mice. LC–MS revealed CsA restricted the excessive activation of neutrophils by inhibiting the PPP metabolism in neutrophils isolated from human peripheral, at the same time, the activity of G6PD (rate-limited enzyme of PPP) decreased. CsA downregulated PPP metabolism and decreased NETs release by inhibiting the expression of P53. The RNA-seq suggested altered gene expression in neutrophils by CsA, P53 was found to interact with G6PD the most tightly, and the increased P53 protein reduced the G6PD activity and ROS generation in the PPP, thus inhibiting the excessive activation of neutrophils. Taken together, our study demonstrated that CsA could lower the activity of the PPP rate-limiting enzyme G6PD and inhibit a large number of ROS produced through intracellular PPP metabolism (specifically by down-regulating NADPH/NADP +) by activating P53 protein, thus reducing the formation of NETs and alleviating colitis.

Previous research has observed that NETs could modulate adaptive immunity by interacting with DC and T cells in autoimmune diseases like SLE and asthma (Garcia-Romo et al. 2011; Krishnamoorthy et al. 2018). Recent evidence indicates that NETs also play an essential and significant role in the pathogenesis of IBD and it has also been found that the potential of NETs release from peripheral blood and NETs expression was upregulated in patients with IBD, no matter whether the disease was active (Dos Santos Ramos et al. 2021). Besides that, it also showed that the NETs enrichment score could differentiate mild to moderate-to-severe activity (Haberman et al. 2019). Our results certified that NETs enrichment was elevated in patients with active and inactive UC, which was consistent with general studies (Dinallo et al. 2019; Li et al. 2020). Although our study has found the vital role of NETs in UC, especially in patients with relatively severe stages, the influence of NETs on innate and adaptive immunity in UC was still worthy of further investigation.

It has been well accepted that CsA inhibited T cells' function by affecting the nuclear factor of activated T cells (Liu et al. 1991), nevertheless, the new findings indicate that CsA also affects innate immune cells, including DCs, macrophages, and neutrophils (Hawkshaw and Paus 2021; Liddicoat and Lavelle 2019). There was study found that only 4%-9% CsA was distributed in T cells, while neutrophils consisted of 5%-12% CsA in vivo (Flores et al. 2019). Furthermore, CsA treated patients with severe UC efficiently, which had a close connection with neutrophils instead of T cells (Friedrich et al. 2021). For another, neutrophils modulated by CsA successfully in patients who responded to this therapy (Lu et al. 2021). The mechanism by which DCs or macrophages induce and inhibit IL-2 is similar to that of T cells. When the pathogen stimulates, the extracellular Ca^2+^ influx and calcineurin are activated. Calcineurin dephosphorylates nuclear factor of activated T cells (NFAT), which transfers it to the nucleus and supports the transcription of inflammatory genes. CsA inhibits the expression of NFAT-dependent genes by forming complexes with cyclophilin A (Vandewalle et al. 2014). Only a few studies have reported the regulation of calcineurin-NFAT pathway in neutrophils and one study found that CsA could balance neutrophil immune functions via SIRT6 by increasing expression of HIF-1α but not the activity of NFAT (Lu et al. 2021). Our study illustrated that the expression of NETs-related proteins decreased in the colon from the colitis model treated by CsA. However, CsA also downregulated the transcriptional levels of TNF-α and IL-1β, which could induce NETs release, according to a published study (Keshari et al. 2012). Thus, reduced NETs expression treated by CsA could be through direct and indirect ways.

Previous research has found that CsA downregulated NETs release, which was related to the change of intracellular Ca^2+^ flux (Zhang et al. 2022). Our study showed that CsA with decreasing ROS level could reduce the expression of NETs stimulated by LPS. In response to stress, the metabolism in neutrophils has changed to adapt to the circumstance (Injarabian et al. 2019).To supplement, it has been reported that glycolysis was important for NETs release, which was inhibited in the presence of 2-DG (Rodríguez-Espinosa et al. 2015). It was also recognized that suppressing PPP inhibited oxidative burst and dampened NETs release (Azevedo et al. 2015). In our study, we found that products from PPP were reduced, while metabolite from glycolysis increased in neutrophils stimulated by LPS after treatment of CsA. The activity of G6PD was upregulated in neutrophils from patients with UC and downregulated by CsA under the stimulation of LPS. Moreover, 6-AN was utilized to certify further that effect of CsA on NETs release was dependent on PPP. Although ATP, which was mainly produced via glycolysis and ROS which was originated from PPP in majority were both crucial to ROS-dependent NETs release (Amini et al. 2018; Azevedo et al. 2015), there was intersection point where the products of PPP entered into glycolysis with less effect on production of ATP (Jiang et al. 2014). Hence, PPP was the critical pathway in regulating the activation of neutrophils. Recently, G6PDi, the inhibitor of G6PD, was developed and found to inhibit the excretion of interferon-γ and TNF-α from T cells (Ghergurovich et al. 2020), representing that PPP metabolism had an essential role on other immune cells and was waiting to investigate.

Consequently, our study explored the possible intrinsic mechanism of regulating the activity of G6PD by CsA. It has been proved that P53, a tumour suppressor, was shown to regulate metabolism, including glycolysis and oxidative phosphorylation. Moreover, P53 suppressed glucose consumption, NADPH production and biosynthesis via the PPP (Kondoh et al. 2005; Matoba et al. 2006; Vousden and Ryan 2009). P53 reduce the activity of Glucose-6-phosphate dehydrogenase (G6PD), the first rate-limiting enzyme in PPP metabolism, by binding to G6PD and preventing the formation of its active dimer (Jiang et al. 2011). From STRING, it was found that P53 could be combined with G6PD and inhibit the activity directly. Our study showed that the inhibiting effect of CsA on G6PD activity could be reversed by the P53 inhibitor, which means that the influence of CsA on G6PD activity might have a link with P53 related pathway. Nonetheless, it has been reported that P53 could hydrolyze fructose-2;6-bisphosphate to inhibit glycolysis via induction of TP53-induced glycolysis regulatory phosphatase (TIGAR) in cancer cells line (Bensaad et al. 2006). However, whether the expression of TIGAR changed in neutrophils under activation was still unknown. As described above, the overall alteration of glucose metabolism in neutrophils needs to be studied in future.

There were several limitations in our research. On the one hand, the only visualized thing in the colitis model was decreased NETs release in the CsA-treated group without the influence of NETs on the severity of colitis and possible reason. Moreover, whether this phenomenon is due to neutrophils in UC releasing NETs more easily or there are more neutrophils in UC tissues has not been sufficiently studied. PAD^−/−^ mice or DNase injection could be utilized, and fluorescein-labelled neutrophils should be applied to explore the state of neutrophils in inflammation-involved tissue in the future. On the other hand, it was better to use metabolic flux to reflect the complete change of intracellular metabolism, including glucose and glutamine. Last, more P53 mutants were observed in inflammatory tissue from patients with UC (Hussain et al. 2000; Lu et al. 2017). It has also been proved that P53 mutants lost the function of inhibition of G6PD, particularly R175H, R273H and G279E (Jiang et al. 2011). Therefore, exact mutants of P53 in patients with UC could be examined and explore whether the relationship occurs between P53 mutants and treatment response.

## Conclusions

To summarize, our study elucidated that NETs enrichment had a positive relationship with the severity of UC and demonstrated that CsA downregulated the expression of NETs by decreasing the activity of G6PD and PPP metabolism, which might be via P53 activation.

### Supplementary Information

**Additional file 1: Fig. S1**. The influence of CsA on NETs expression and intracellular ROS level is dependent on P53 activation. **A** Change of intracellular ROS level in neutrophils stimulated by LPS or CsA in the presence of PFT-α detected by fluorescence microplate for 1 h (*P < 0.05, **P < 0.01 for neutrophils treated with LPS + CsA vs LPS + CsA + PFT-α at the time of 40 and 50 min separately). **B** Immunofluorescence and ratio of fluorescence intensity of Sytox green to Hoechst 33342 in neutrophils stimulated by LPS, CsA in the presence of PFT-α (* P < 0.05 for neutrophils treated with LPS + CsA vs LPS + CsA + PFT-α, *** P < 0.05 for neutrophils treated with LPS vs LPS + CsA).

**Additional file 2:** **Table S1.** NETs-related genes.

**Additional file 3:** **Table S2:** Primer sequences used in this study.

## Data Availability

All data supporting the findings of this study are available within the paper and its additional materials.

## Abbreviations

NETs: Neutrophil extracellular traps
UC: Ulcerative colitis
CsA: Cyclosporine A
PPP: Pentose phosphate pathway
ROS: Reactive oxygen species
NE: Neutrophil elastase
citH3: Citrullinated histone h3
