# Supplementary figures and images for: Cyclosporine A alleviates colitis by inhibiting the formation of neutrophil extracellular traps via the regulating pentose phosphate pathway
Source: Mol Med. 2023 Dec 13;29:169. doi: 10.1186/s10020-023-00758-8 (PMC10720086; doi:10.1186/s10020-023-00758-8)

## Slide 1
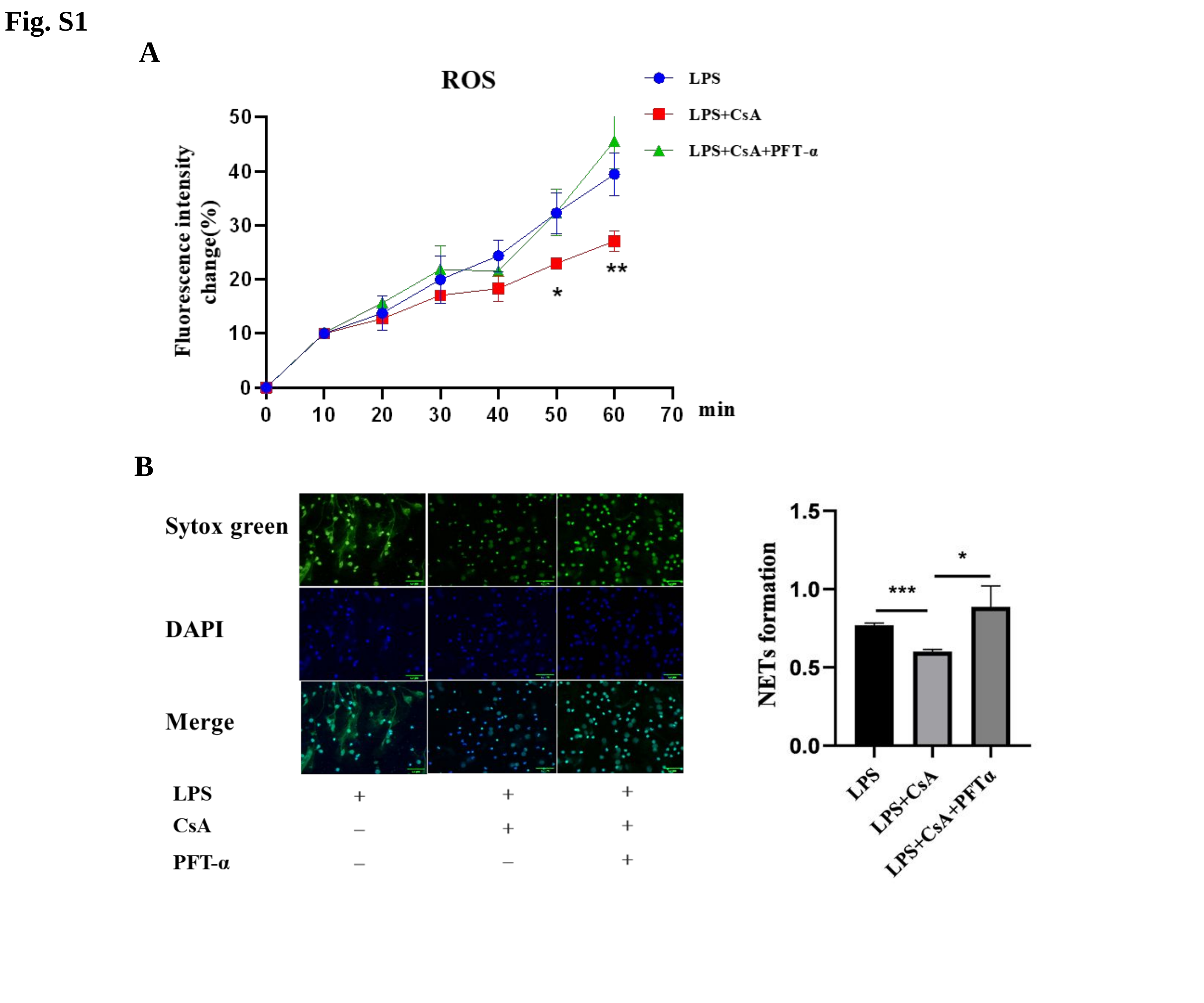

Fig. S1
A
B

Supplement: Supplementary file 1 — Additional file 1: Fig. S1. The influence of CsA on NETs expression and intracellular ROS level is dependent on P53 activation. A Change of intracellular ROS level in neutrophils stimulated by LPS or CsA in the presence of PFT-α detected by fluorescence microplate for 1 h (*P < 0.05, **P < 0.01 for neutrophils treated with LPS + CsA vs LPS + CsA + PFT-α at the time of 40 and 50 min separately). B Immunofluorescence and ratio of fluorescence intensity of Sytox green to Hoechst 33342 in neutrophils stimulated by LPS, CsA in the presence of PFT-α (* P < 0.05 for neutrophils treated with LPS + CsA vs LPS + CsA + PFT-α, *** P < 0.05 for neutrophils treated with LPS vs LPS + CsA). [file 10020_2023_758_MOESM1_ESM.pptx]
