# Supplementary material for: Cyclosporine A alleviates colitis by inhibiting the formation of neutrophil extracellular traps via the regulating pentose phosphate pathway
Source: Mol Med. 2023 Dec 13;29:169. doi: 10.1186/s10020-023-00758-8 (PMC10720086; doi:10.1186/s10020-023-00758-8)
Supplement: Supplementary file 3 — Additional file 3: Table S2: Primer sequences used in this study. [file 10020_2023_758_MOESM3_ESM.docx]

| **Table S2.** Primer sequences used in this study | | |
| --- | --- | --- |
| **Gene name** | **F/R** | **Sequences 5'-3'** |
| IL-1β | F | GAAATGCCACCTTTTGACAGTG |
|  | R | TGGATGCTCTCATCAGGACAG |
| TNF-α | F | CTGAACTTCGGGGTGATCGG |
|  | R | GGCTTGTCACTCGAATTTTGAGA |
| G6PD | F | CGAGGCCGTCACCAAGAAC |
|  | R | GTAGTGGTCGATGCGGTAGA |
| TP53 | F | GAGGTTGGCTCTGACTGTACC |
|  | R | TCCGTCCCAGTAGATTACCAC |
| Actb | F | ATTGCCGACAGGATGCAGAA |
|  | R | GCTGATCCACATCTGCTGGAA |
